# Beware of host immune responses towards bacteriophages potentially impacting phage therapy

**DOI:** 10.1186/s13567-025-01600-1

**Published:** 2025-08-15

**Authors:** Thomas Démoulins, Jérémy D. R. Cherbuin, Thatcha Yimthin, Lukas Eggerschwiler, Fazal Adnan, Joerg Jores

**Affiliations:** 1 Institute of Veterinary Bacteriology, Department of Infectious Diseases and Pathobiology, Vetsuisse Faculty, University of Bern, Bern, Switzerland; 2 Graduate School for Cellular and Biomedical Sciences, University of Bern, Bern, Switzerland; 3 Research Contracts Animals Group, 1725 Agroscope, Posieux, Switzerland; 4 Atta Ur Rahman School of Applied Biosciences (ASAB), National University of Sciences and Technology (NUST), Islamabad, 44000 Pakistan; 5 Multidisciplinary Center for Infectious Diseases (MCID), University of Bern, Bern, Switzerland

**Keywords:** Bacteriophages, ex vivo immune response, phage therapy, cattle

## Abstract

The rising spread of antimicrobial resistance challenges animal welfare, productivity and food supply with respect to bacterial infections in different livestock species. Bacteriophages offer alternative means to control infections with resistant bacteria. This work investigated the host immune responses towards lytic bacteriophages K and T1, acting respectively on Gram-positive or Gram-negative bacteria. We employed an ex vivo platform to decipher various immune responses elicited by primary blood cells of eight outbred cattle. Bacteriophage K was not recognized whereas bacteriophage T1 induced a profound immune response. Therefore, prior characterization of host immune responses towards therapeutic phages should be considered before therapy starts.

**Supplementary Information:**

The online version contains supplementary material available at 10.1186/s13567-025-01600-1.

## Introduction, methods and results

Control of bacterial infections cannot rely exclusively on antimicrobials due to increasing resistance and little incentives of pharmaceutical companies to develop new classes of antimicrobials that come with huge costs. As a result, the development pipeline for novel antimicrobials dried up. Besides the development of next generation vaccines, phage therapy appears as a promising complementary treatment path for infections with bacterial pathogens [1]. First, the diffusion of bacteriophages in different organs was observed in mice after injection [2–4]. Moreover, oral administration of phages in human patients proved to be safe [5–7]. Logically, the potential interaction of phages with the host immune system have gained interest a decade ago, showing in mice a weak—but detectable—immune response and antibody production against a T4 coliphage in the gut and blood [8]. Immunostimulatory properties of phages on human cells were further reported for T4 tail adhesin, *Staphylococcus* phage K and five *Pseudomonas* phages, without inducing toxic or antiproliferative effects [9–11]. However, the specific interaction of bacteriophages with livestock-derived immune cells such as ruminant peripheral blood mononuclear cells (PBMCs) remains a gap of knowledge. Bacteriophages are viruses and like other microorganisms have the capacity to induce innate as well as memory immune responses. This matters since the latter immune responses are likely to attack the phages and contribute to their clearance, which depending on the delay, can be either negative (early phage elimination before they had time to kill bacteria) or positive (late phage elimination once they have killed bacteria).

Consequently the host immune response can negatively impact the phage therapy twofold by producing antibodies directed towards phage particles that assist in phage clearance [12] or by causing side effects such as aggravated intestinal inflammation and colitis which was reported for germ-free mice [13]. Therefore, a synergistic approach excluding existing negative host immune responses in addition to the confirmation of the phage capacity to lyse bacterial target cells, will foster successful phage-assisted treatment [14, 15]. In the present study, the ex vivo immune response towards two well characterized lytic phages (coliphage T1 and *S. aureus* phage K) was carried out for the first time in bovine primary blood cells.

In line with the 3R principles, we recently developed an ex vivo laboratory platform employing PBMCs of outbred animals to investigate bovine-pathogen interactions [16]. Blood of *Bos taurus* type Holstein Friesian cows (aged 1–3 years) was collected at the Agroscope research facility (Posieux, Switzerland). The donor cattle enrolled in the assays were outbred animals and represent herds that are not mingling with other animals outside the facilities.

Application of multiparameter flow cytometry assay and multiplex immunoassay have been described recently [16], apart from very few changes of antibodies and fluorophores, namely home-made conjugation of CD3 employed Zenon Alexa Fluor 568 labelling kit (instead of Alexa Fluor 532 Antibody Labeling Kit), and newly used CD45RO (clone IL-A116, instead of CD44).

Production strains for phages T1 and K were *E. coli* DH5α and * S. aureus* PS58, respectively. *E. coli* DH5α was grown in Luria–Bertani broth (LB) medium and *S. aureus* PS58 in Tryptic soy broth (TSB) medium at 37 °C. Stocks of bacteriophages were produced in liquid culture. Briefly, each phage was added at a multiplicity of infection 1 to a 5 mL culture of the production strain in exponential growth phase determined via measurement of optical density at OD_600_.

**Figure 1:**
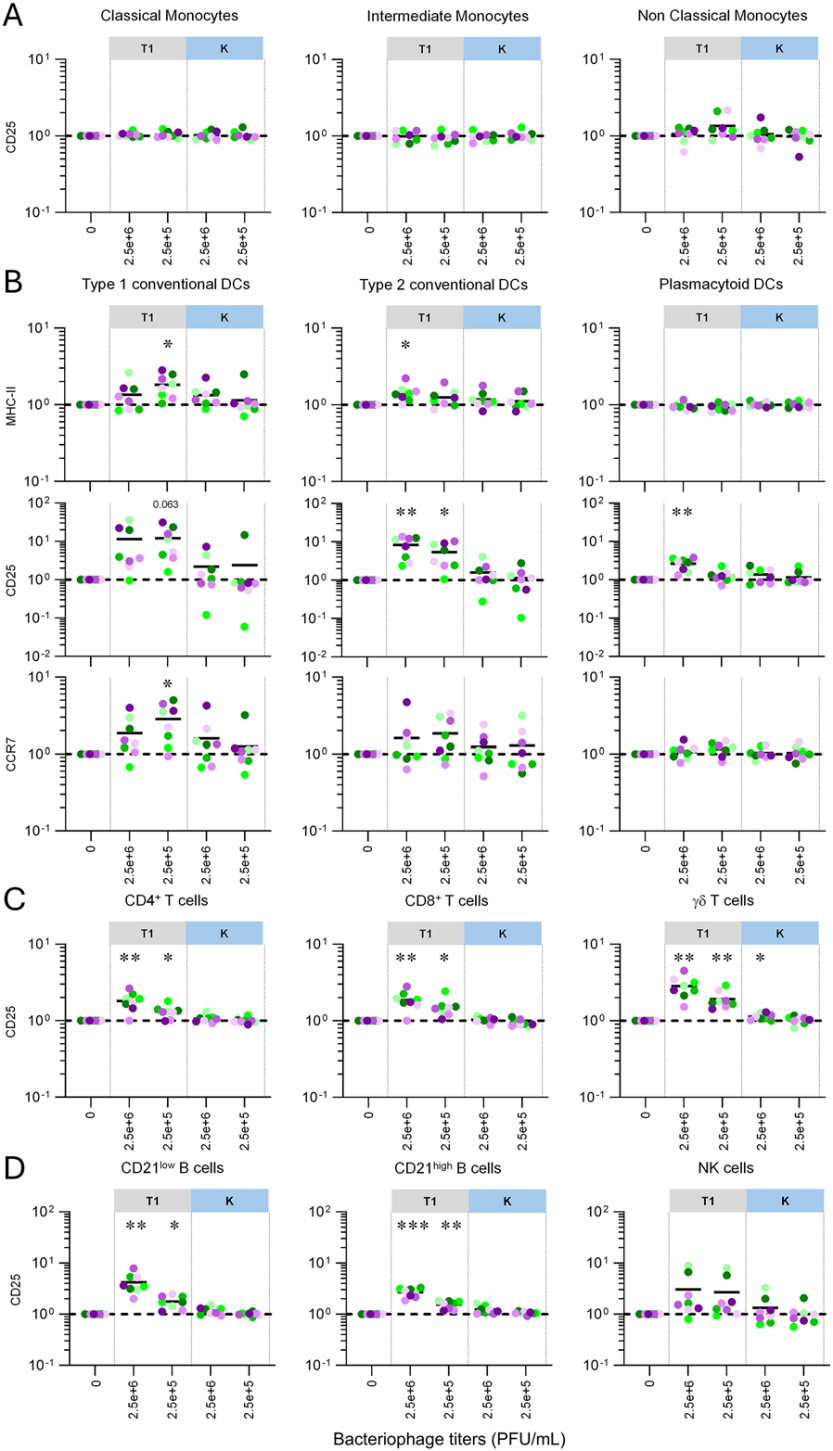
**Stimulation of primary blood cells by T1 and K bacteriophages under physiological temperature.** PBMCs from 8 individual animals per group, were either let for 48 h unstimulated (reference points for the assay, “0”), stimulated with T1 (grey color) or with K (blue color). The fold changes analysis of activation/maturation markers was determined by FCM with FlowJo. Cells from the individual cattle are represented by separate symbols; for stimulated samples, mean fluorescence intensity (MFI) values are normalized to that obtained with the reference point from the same animal. Results obtained for Monocyte (**A**), Dendritic cell (**B**), T-cell (**C**), B-cell and NK-cell (**D**) subsets. Statistical analysis was done using the GraphPad Prism 8 software (GraphPad software, La Jolla, CA, USA). To determine differences between groups, one-way repeated measure ANOVA followed by Geisser-Greenhouse correction were used, as appropriate. (*p < 0.05, **p < 0.01, ***p < 0.001).

**Figure 2:**
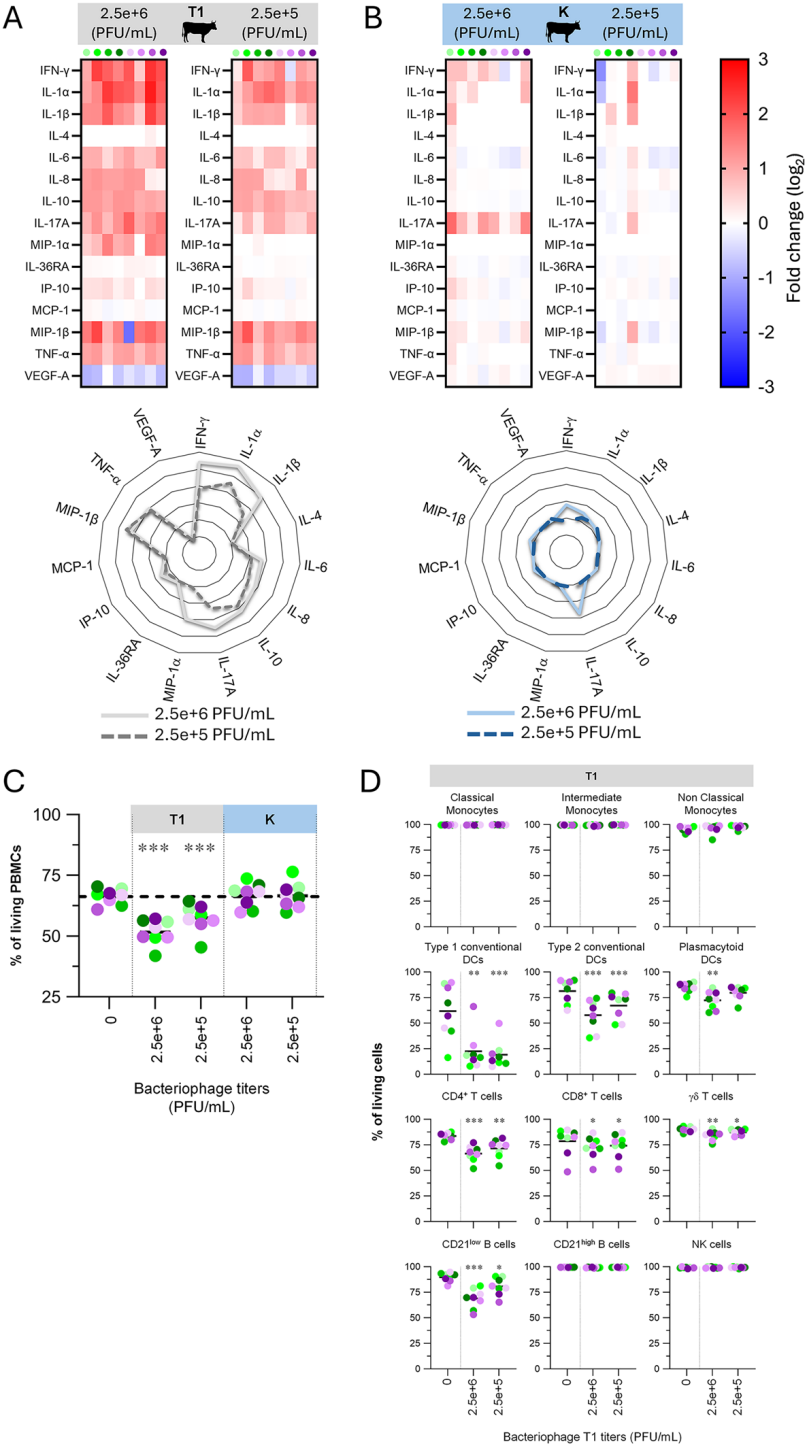
**Induction of cytokines by primary blood cells following exposure to T1 and K bacteriophages under physiological temperature.** PBMCs from 8 individual animals per group were either for 48 h unstimulated or stimulated at ruminant body temperature (38.5 °C). **A** Induction of cytokines following exposure to T1 bacteriophage. Upper panel: a single measurement was done per sample tested, and each symbol represents an individual cow. Heat map shows log_2_-fold changes in concentration of 15 cytokines/chemokines. For a given cytokine/chemokine, normalization was as follow: [concentration for a given animal]/[average concentration of reference points]. Lower panel: Radar plots showing the mean of 8 animals of log_2_-fold changes in concentration. **B** Same as (**A**), but for K bacteriophage. **C** Impact of bacteriophage exposure on viable cell frequencies. A primary gate (P1) was set on FSC-A versus SSC-A, then, the percentage of living PBMCs (negative for Live/Dead marker) was quantified by FCM. Each symbol represents an individual animal and is the average of three independent measurements (“Antigen presenting cells”, “T cells”, and “B cells, NK cells” panels). **D** Impact of exposure to T1 bacteriophage on viable immune cell subset frequencies. The percentage of living cells for a given subset (negative for Live/Dead marker) was quantified by FCM. Statistical analysis was done using the GraphPad Prism 8 software (GraphPad software, La Jolla, CA, USA). To determine differences between groups, one-way repeated measure ANOVA followed by Geisser-Greenhouse correction were used, as appropriate. (* *p* < 0.05, **p < 0.01, ***p < 0.001).

In the first experiment (Figures 1 and 2), no cesium chloride (CsCl) gradient ultracentrifugation was performed for better bacteriophage purity.

**Figure 3:**
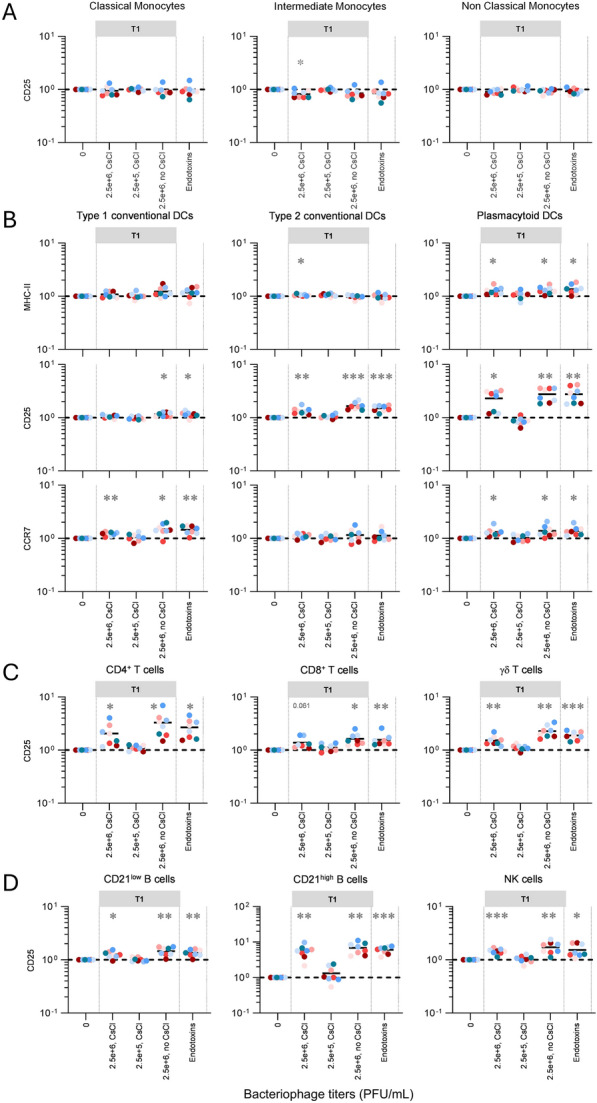
**Stimulation of primary blood cells by T1 bacteriophages purified with cesium chloride gradients.** PBMCs from 8 individual animals per group, were either let for 48 h unstimulated (reference points for the assay, “0”), stimulated with T1 (grey color) or with standard endotoxin solution as positive control. The fold changes analysis of activation/maturation markers was determined by FCM with FlowJo. Cells from the individual cattle are represented by separate symbols; for stimulated samples, mean fluorescence intensity (MFI) values are normalized to that obtained with the reference point from the same animal. Results obtained for Monocyte (**A**), Dendritic cell (**B**), T-cell (**C**), B-cell and NK-cell (**D**) subsets. Statistical analysis was done using the GraphPad Prism 8 software (GraphPad software, La Jolla, CA, USA). To determine differences between groups, one-way repeated measure ANOVA followed by Geisser-Greenhouse correction were used, as appropriate. (*p < 0.05, **p < 0.01, ***p < 0.001). CsCl: Cesium Chloride.

**Figure 4:**
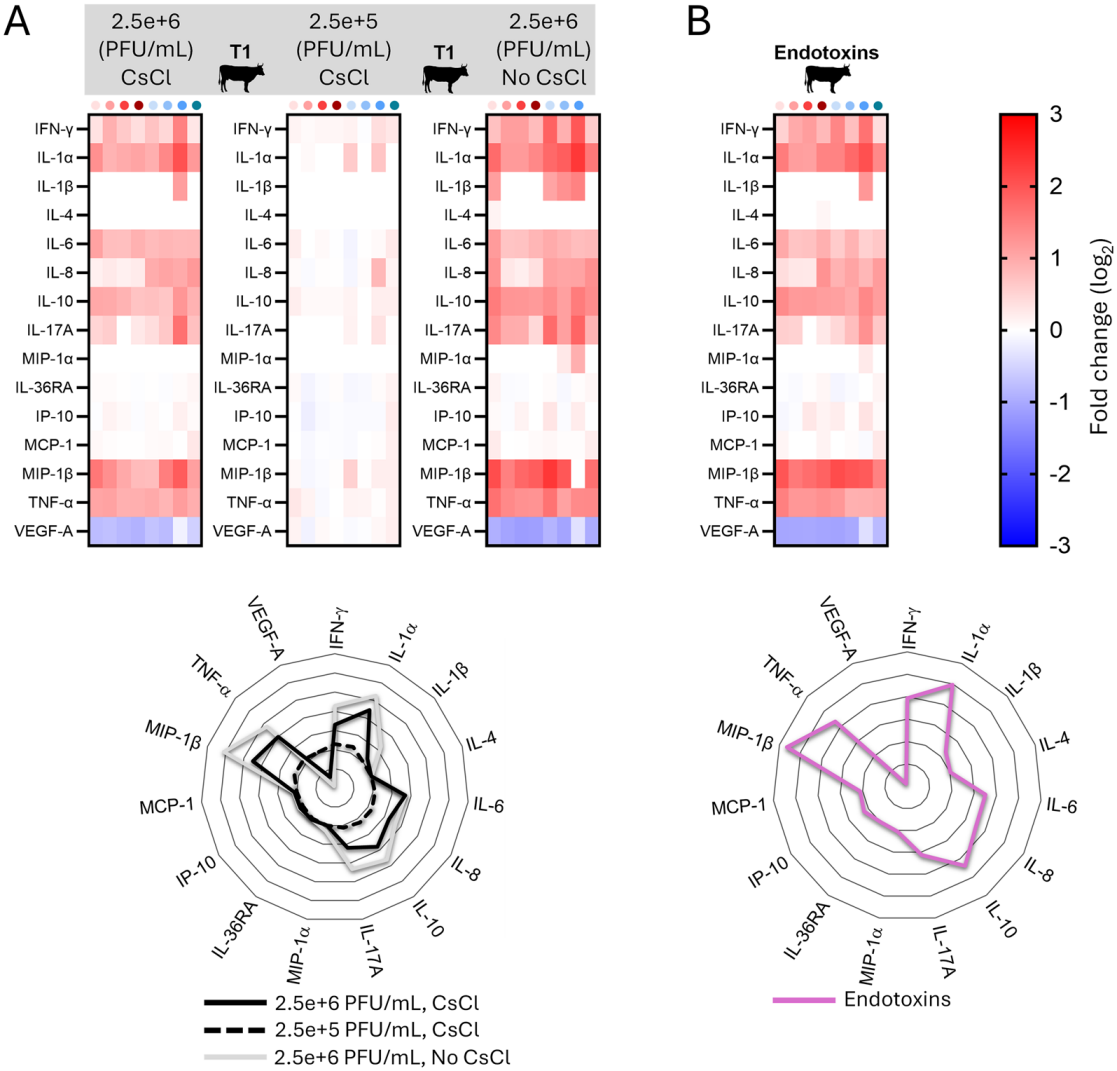
**Induction of cytokines by primary blood cells following exposure to T1 bacteriophages purified with cesium chloride gradient**. PBMCs from 8 individual animals per group, were either let for 48 h unstimulated (reference points for the assay), stimulated with T1 (grey color) (**A**) or with standard endotoxins solution as positive control (**B**). A single measurement was done per sample tested, and each symbol represents an individual cow. Upper panel: Heat map shows log_2_-fold changes in concentration of 15 cytokines/chemokines. For a given cytokine/chemokine, normalization was as follow: [concentration for a given animal]/[average concentration of reference points]. Lower panel: Radar plots showing the mean of 8 animals of log_2_-fold changes in concentration. CsCl: Cesium Chloride.

In the second experiment (Figures 3 and 4), following bacterial lysis, confirmed by OD_600_ measurements, phages were purified by CsCl gradient ultracentrifugation as previously described elsewhere [17], which also served as the first step in a two-step endotoxin removal process. Samples were transferred to ultra-clear centrifuge tubes (14 × 89 mm, Beckman & Coulter, Inc., USA) and centrifuged in a SW41 Ti rotor (Beckman & Coulter) using a HITACHI CP100NX ultracentrifuge at 100 000 × *g* at 4 °C for 2 h. Subsequently supernatants were discarded, and phage-containing pellets were resuspended in SM buffer.

Finally, before testing them in our ex vivo platform, all phage lysates were further purified using the “EndoTrap^®^ HD 5/1” kit (LIONEX GMBH, Germany) following manufacturer instructions. Next, endotoxin quantification was done using the “Pierce™ Chromogenic Endotoxin Quant Kit” (Thermo Fisher Scientific) with the high standards methods and strictly following manufacturer instructions. Finally, bacteriophage enumeration was done using the double agar overlay plaque assay method as previously described [18].

We first assessed the responsiveness of PBMCs to bacteriophages and determined the titers to be used: 2.5e + 6 PFU/mL (corresponding to 5 phage particles per 100 blood cells, “High” titer); 2.5e + 5 PFU/mL (corresponding to 5 phage particles per 1000 blood cells, “Low” titer). We next performed an in-depth investigation of immune cell subset activation state following phage exposure. If monocyte subsets did not response to both bacteriophages (Figure 1A), dendritic cells (DCs) were clearly activated by bacteriophage T1, as witnessed by the upregulation of MHC-II (Type 1 and 2 cDCs), CD25 (Type 1 and 2 cDCs, pDCs) and CCR7 (Type 1 cDCs); in contrast bacteriophage K proved to have almost no effect (Figure 1B). Finally, bacteriophage T1 positively modulated the activation levels of all T- and B-cell subsets (strong CD25 upregulation), whereas bacteriophage K played almost no role (with the slight exception of γδ T cells) (Figures 1C and D).

In the meantime, supernatants were collected and investigated for cytokine and chemokine secretion. Upon stimulation with high titer of bacteriophage T1, the most noticeable result consisted of elevated levels of a set of pro-inflammatory cytokines, namely IL-1α, IL-1β, IL-6, MIP-1α (= CCL3), MIP-1β (= CCL4), and TNF-α, as well as pro-Th1 (IFN-γ) and pro-Th17 (IL-17). As expected, low titer exhibited a similar trend in cytokine induction with a clear reduction in overall cytokine levels, as illustrated in Figure 2A. Of note, cytokine induction profiles were quite superimposable when analysis was done for sample stimulated with bacteriophages before purification on column, except MIP-1β (Additional file 1).

Again, the above results obtained for bacteriophage T1 did not apply to bacteriophage K, which only led to moderate induction of IL-17, revealing clear divergent influence of both viruses on ruminant immune system (Figure 2B). To confirm the stronger interaction of bacteriophage T1 with bovine immune cells, a viability assay was conducted. Effectively, exposure to bacteriophage T1 resulted in a marked reduction of the frequency of living PBMCs, whether bacteriophage K had no effect (Figure 2C). When this analysis was applied to individual immune cell subsets, the viability of monocytes, CD21^high^ B cells and NK cells proved to remain unchanged following stimulation by bacteriophage T1, whereas all other subsets showed a strong decrease of percentage of living cells, with the Type 1 cDCs being particularly affected (Figure 2D).

At this stage however, without endotoxin level measurement, we still had the theorical possibility that the ex vivo response following bacteriophage T1 exposure was raised by residual endotoxins originating from Gram-negative *E. coli* (unlike bacteriophage K, whose production occurred in endotoxin-free conditions due to Gram-positive *S. aureus*). To rule out this possibility, a new batch of bacteriophage T1 was produced, but this time using CsCl gradient purification followed by a round of endotoxin purification steps for optimal concentration and purification. The levels of endotoxin of each phage production were measured and showed that both bacteriophages T1 and K were free of endotoxin (Additional file 2). For blood cell stimulation, unpurified T1 phages (suspected to contain low amounts of endotoxins) and standard endotoxin solution (high concentrations) were employed as positive controls. As mentioned previously, monocytes hardly responded to any of stimulations by bacteriophages; the same occurred with standard endotoxin solution (Figure 3A). However, bovine PBMCs were still strongly stimulated when exposed to bacteriophage T1 with complete removal of potential residual endotoxins [DCs (Figure 3B), T cells (Figure 3C), B and NK cells (Figure 3D)]. From this, it was evident that bacteriophages T1 themselves had a strong immunostimulatory effect on bovine primary blood cells. Although the activation of DCs was likely to be triggered by the recognition of conserved viral antigens, the CD25 upregulation detected in T and B cells was intriguing. To check whether this activation resulted from a direct or indirect effect, we stimulated sorted T cells (CD4^+^ and CD8^+^) with bacteriophage T1. This offered the opportunity of analyzing the T cells without the presence of other immune cell subsets. CD25 upregulation of sorted T cells was compared with values obtained in T cells from same animals among PBMCs (previous conditions), or sorted T cells from same animals with adding back the negative fraction of the magnetic sort (“Neg. frac. + T cells”, to subtract potential CD25 upregulation caused by the sorting procedure itself). From this experiment, it was clear that T cells were unable to respond directly on their own to bacteriophage T1 (at least CD3^+^ and CD4^+^ T cells), and that previously observed activation in Figures 1 and 3 for this adaptive immune cell subset was due to a bystander effect (Additional file 3).

Again, it was considered important to corroborate the above results with independent readout, which is why the cytokine secretion by PBMCs was then evaluated. Exposure to bacteriophage T1 purified with CsCl gradient still promoted a balanced cytokine response, consisting in a set of pro-inflammatory, pro-Th1, pro-Th17 and anti-inflammatory (IL-10) cytokines, somehow comparable to what detected for unpurified T1 phage (Figure 4A) or standard endotoxin solution (Figure 4B).

Lastly, we assessed bacteriophage “viability” after co-incubation with PBMCs. To this aim, titers were assessed before exposure and at the end of the 48 h exposure to PBMCs, using classical plaque assay method: no significant reduction in phage titer was observed between the two time points with either high or low titers, bacteriophage T1 or K (Bacteriophage T1, Additional file 4; Bacteriophage K, Additional file 5), compared to their respective controls (phage lysates incubated in a saline buffer). Interestingly, we even observed lower phage titers in controls, supporting the notion that phage integrity remains stable in close contact to bovine blood cells.

Overall, our results showed differential impacts between two bacteriophages on bovine immune cells. Notably, T1 strain had a clear immunostimulatory effect that must be considered before initiating any T1-based therapy.

## Discussion

The foreign nature of bacteriophages makes them susceptible to activate the innate immune system via danger signals known as pathogen-associated molecular patterns (PAMPs) and damage-associated molecular patterns (DAMPs). Additionally, their particulate shapes can promote uptake by antigen presenting cells (APCs), which in turn activate the adaptive immune system (for a review see [19]). The combined activation of both innate and adaptive arms of the immune system meets all the requirements to induce robust and long-lasting responses in cattle. Effectively, what was already described in humans or mouse proved to apply as well to ruminants, with the stimulation of most immune cell subsets by bacteriophage T1 (apart from monocytes). To our knowledge, our work provides for the first time evidence that some bacteriophage candidates can induce a strong ex vivo immune response in cattle. Whether these immunomodulatory properties would be beneficial (adjuvating the immune response towards bacterial agent) or detrimental (immune response against the phage itself, immune cell exhaustion) administrated in vivo in cattle requires further investigations. In line with this consideration, bacteriophage T1 induces on one hand a strong immune response, but on the other hand its titer remains high following co-incubation with PBMCs, not ruling out that phage therapy using this specific strain, if ultimately beneficial, would have long-lasting effect. We provide a framework platform that will assist future studies and treatments on other bacteriophages to control for example *E. coli* O157:H7 foodborne infections (for a review see [20] or [21]) or on phage cocktails developed for future treatment of bovine mastitis induced by *S. aureus* [22, 23].

Herein, we choose bacteriophage K because of its rather large host-range [9], and bacteriophage T1 because of its efficiency against *E. coli* O157:H7 strains. The latter is of importance, because adult cattle do not present any disease with this serotype, they are considered the primary reservoir, with potential outcome on human health [24]. The morphological differences between those two bacteriophages, K being a myovirus [25] while T1 a siphovirus [26], was expected to interact differently with bovine host cells and thus to induce differential ex vivo immune responses. Effectively, T1 and K bacteriophages varied in their intrinsic immunogenicity (strong for T1, very low for K) probably because immune cells of the cattle had a different history with respect to interactions with these or closely related bacteriophages with similar epitopes. The identification of the reasons of differential responses is out of scope of the present study. Altogether, the present results make a case to study existing responses before phage therapy gets initiated. However, the use of PBMCs to monitor phage-related immune responses has limitations. Ultimately, in vivo experiments reflect best the complex host-phage interactions and the global impact on the immune system. Effectively, the development of an immunological memory would imply cell migration to draining lymph nodes, and this could not be evaluated in the present study.

Overall, our study delivered important insights in potential undesired immune responses that might affect phage therapy in ruminants. Above all, this work shows that: (i) envisaged bacteriophages for therapy in the veterinary field should be carefully assessed ex vivo for their potential interaction with host immune cells; (ii) the immunomodulation outcome of a bacteriophage is not straightforward, but highly dependent on its morphology. Given the success of personalized medicine such as phage therapy in the human field, we see great prospects for personalized phage applications in veterinary medicine. This applies not only to large animal models but also to high value animals and the poultry sector.

## Supplementary Information

**Additional file 1.** **Induction of cytokines by primary blood cells following exposure to bacteriophage T1 purified or not on column.** PBMCs from 8 individual animals per group were either for 48 h unstimulated or stimulated at ruminant body temperature (38.5 °C). A single measurement was done per sample tested, and each symbol represents an individual cow. Upper panel: heat map showing log_2_-fold changes in concentration of 15 cytokines/chemokines; for a given cytokine/chemokine, normalization was as follow: [concentration for a given animal]/[average concentration of reference points]. Lower panel: radar plots showing the mean of 8 animals of log_2_-fold changes in concentration.

**Additional file 2. Measurement of endotoxin levels in the different phage preparations.** **A** Establishment of a standard linear regression curve using endotoxin standard solution of 0, 0.1, 0.25, 0.5 and 1 EU/mL. **B** Measurements in duplicate of all phage preparation. T1 lysate production was diluted to 10^4^ and 10^5^ to measure endotoxin solution present in the stock solutions.

**Additional file 3. Stimulation of isolated T cells by T1 bacteriophages purified with cesium chloride gradients.** PBMCs were prepared as in Figures 1-4, after what T cells were isolated using anti CD4 and CD8 antibodies and Anti-Mouse IgG2a + b MicroBeads (Miltenyi Biotec). PBMCs, sorted T cells, or sorted T cells with adding back the negative fraction (“Neg. frac. + T cells”) were either let for 48 h unstimulated or stimulated with T1 (2.5e + 6 PFU/mL). The fold changes analysis of CD25 expression was determined by FCM with FlowJo. Cells from the individual cattle are represented by separate symbols; for stimulated samples, mean fluorescence intensity (MFI) values are normalized to that obtained with the reference point from the same animal. Statistical analysis was done using the GraphPad Prism 8 software (GraphPad software, La Jolla, CA, USA). To determine differences between groups, one-way repeated measure ANOVA followed by Geisser-Greenhouse correction were used, as appropriate. (*p < 0.05).

**Additional file 4.** **Bacteriophage T1 titers after co-incubation with PBMCs.** Phage titers were determined in triplicates after 24 and 48 h of co-incubation with PBMCs. **A** Unpurified T1 at 2.5e + 6 PFU/mL. **B** Endotoxin-free T1 at 2.5e + 6 PFU/mL. **C** Endotoxin-free T1 at 2.5e + 5 PFU/mL. Phage control stocks of T1 were incubated in SM buffer pH 7.5 (100 mM NaCl, 8 mM MgSO_4_-7H_2_O, Tris-Cl 1 M pH7.5). All tests were performed at ruminant body temperature (38.5 °C).

**Additional file 5. Bacteriophage K titers after co-incubation with PBMCs.** Phage titers were determined in triplicates after 24 and 48 h of co-incubation with PBMCs. **A** Endotoxin-free phage K at 2.5e + 6 PFU/mL. **B** Endotoxin-free phage K titers after coincubation with PBMCs from each animal at 2.5e + 5 PFU/mL. Phage control stocks of K were incubated in SM buffer pH 7.5 (100 mM NaCl, 8 mM MgSO_4_-7H_2_O, Tris-Cl 1 M pH7.5). All tests were performed at ruminant body temperature (38.5 °C).

## Data Availability

All relevant data are included in the manuscript and Additional files.
