# Supplementary figures and images for: Beware of host immune responses towards bacteriophages potentially impacting phage therapy
Source: Vet Res. 2025 Aug 15;56:170. doi: 10.1186/s13567-025-01600-1 (PMC12357382; doi:10.1186/s13567-025-01600-1)

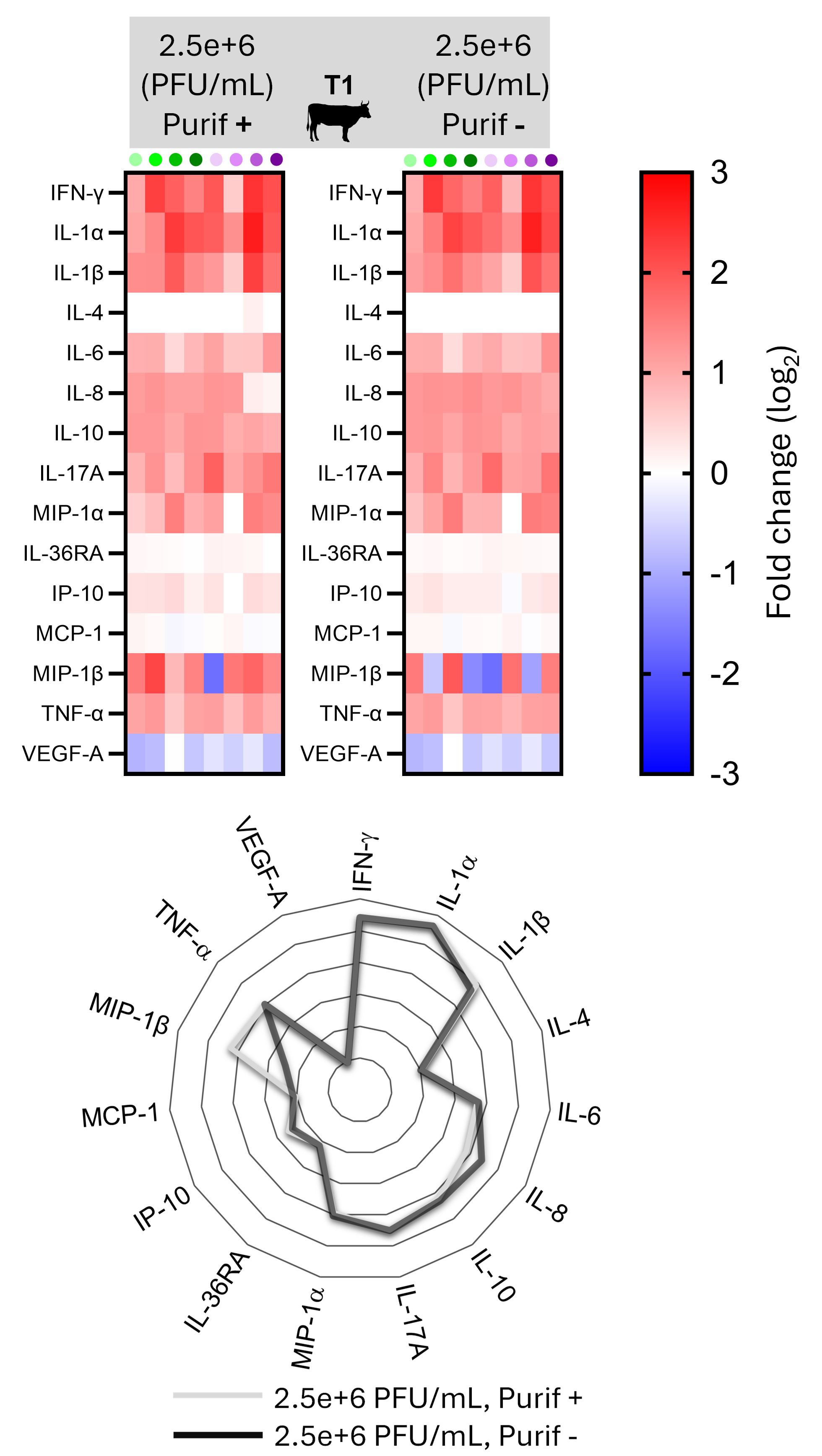

Supplement: Supplementary file 1 — Additional file 1. Induction of cytokines by primary blood cells following exposure to bacteriophage T1 purified or not on column. PBMCs from 8 individual animals per group were either for 48 h unstimulated or stimulated at ruminant body temperature (38.5 °C). A single measurement was done per sample tested, and each symbol represents an individual cow. Upper panel: heat map showing log2-fold changes in concentration of 15 cytokines/chemokines; for a given cytokine/chemokine, normalization was as follow: [concentration for a given animal]/[average concentration of reference points]. Lower panel: radar plots showing the mean of 8 animals of log2-fold changes in concentration. [file 13567_2025_1600_MOESM1_ESM.tif]

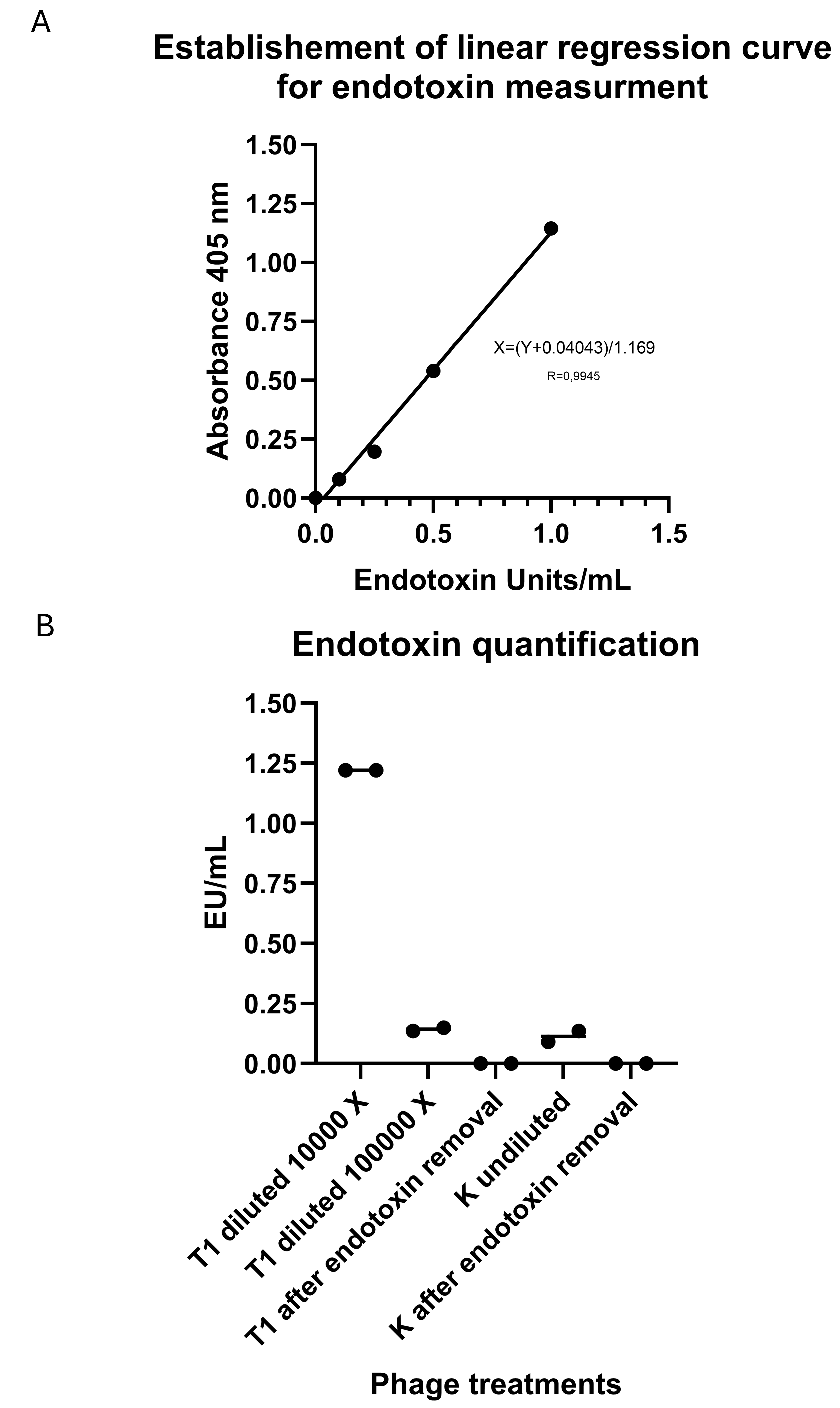

Supplement: Supplementary file 2 — Additional file 2. Measurement of endotoxin levels in the different phage preparations. A Establishment of a standard linear regression curve using endotoxin standard solution of 0, 0.1, 0.25, 0.5 and 1 EU/mL. B Measurements in duplicate of all phage preparation. T1 lysate production was diluted to 104 and 105 to measure endotoxin solution present in the stock solutions. [file 13567_2025_1600_MOESM2_ESM.tif]

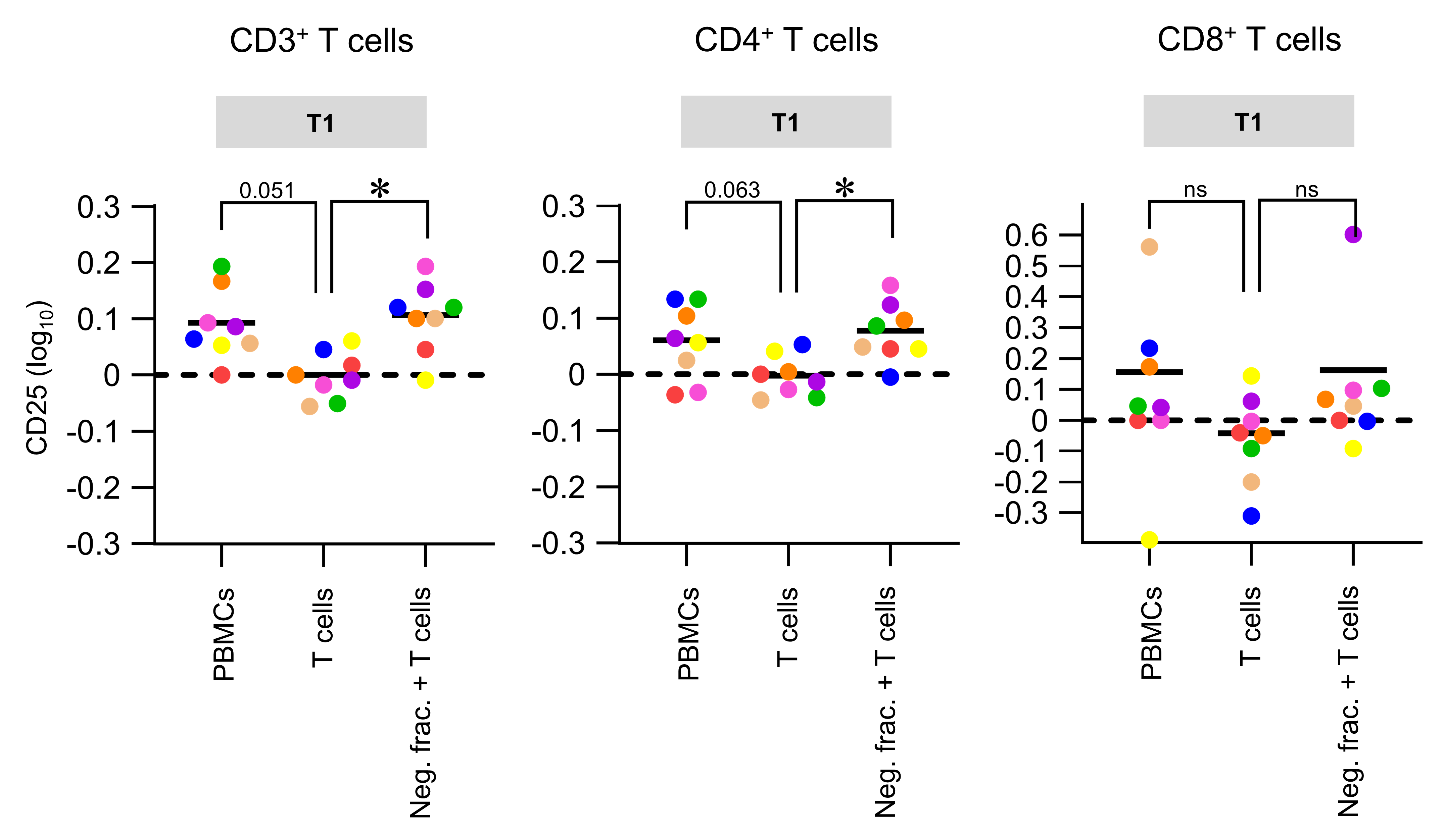

Supplement: Supplementary file 3 — Additional file 3. Stimulation of isolated T cells by T1 bacteriophages purified with cesium chloride gradients. PBMCs were prepared as in Figures 1-4, after what T cells were isolated using anti CD4 and CD8 antibodies and Anti-Mouse IgG2a + b MicroBeads (Miltenyi Biotec). PBMCs, sorted T cells, or sorted T cells with adding back the negative fraction (“Neg. frac. + T cells”) were either let for 48 h unstimulated or stimulated with T1 (2.5e + 6 PFU/mL). The fold changes analysis of CD25 expression was determined by FCM with FlowJo. Cells from the individual cattle are represented by separate symbols; for stimulated samples, mean fluorescence intensity (MFI) values are normalized to that obtained with the reference point from the same animal. Statistical analysis was done using the GraphPad Prism 8 software (GraphPad software, La Jolla, CA, USA). To determine differences between groups, one-way repeated measure ANOVA followed by Geisser-Greenhouse correction were used, as appropriate. (*p < 0.05). [file 13567_2025_1600_MOESM3_ESM.tif]

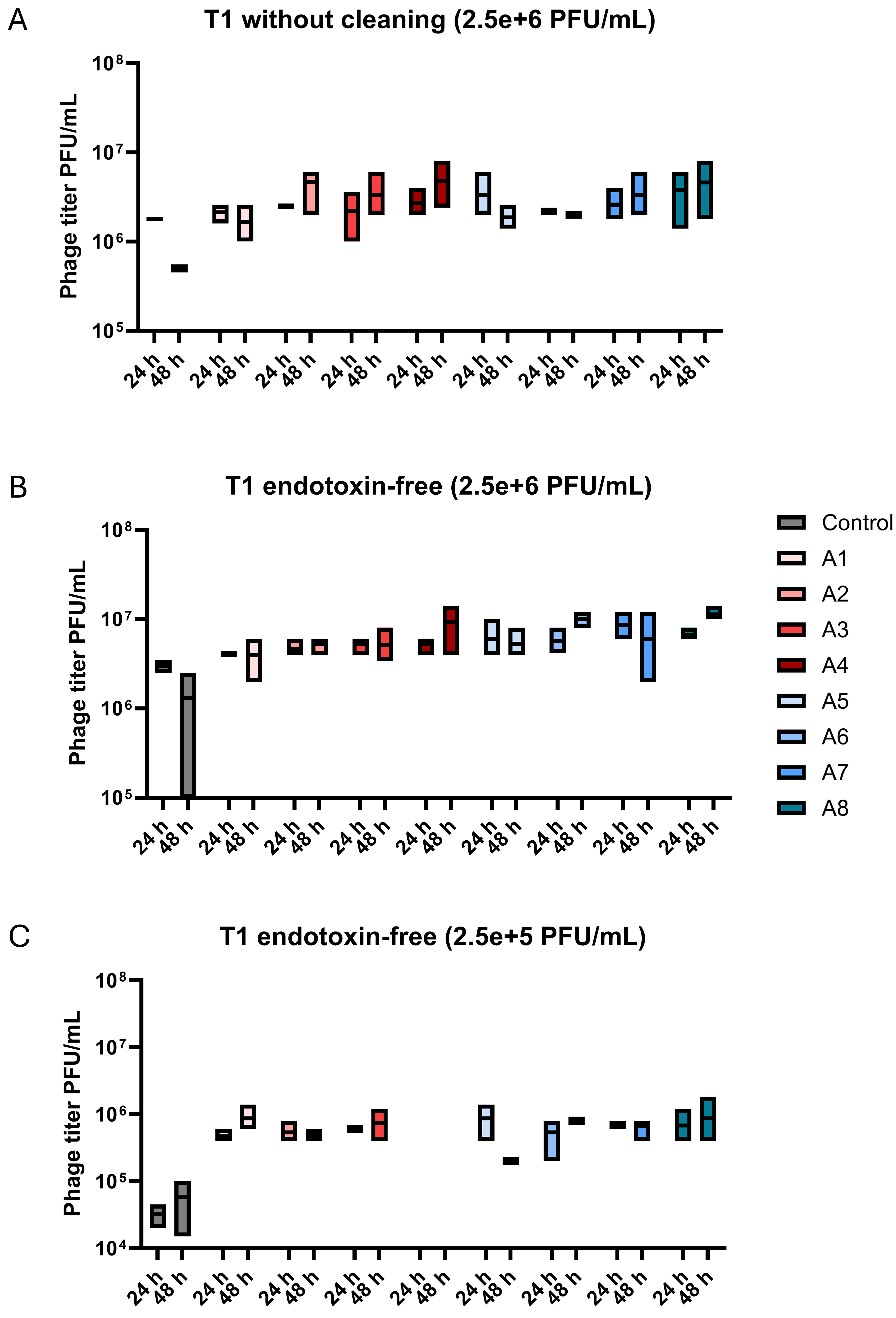

Supplement: Supplementary file 4 — Additional file 4. Bacteriophage T1 titers after co-incubation with PBMCs. Phage titers were determined in triplicates after 24 and 48 h of co-incubation with PBMCs. A Unpurified T1 at 2.5e + 6 PFU/mL. B Endotoxin-free T1 at 2.5e + 6 PFU/mL. C Endotoxin-free T1 at 2.5e + 5 PFU/mL. Phage control stocks of T1 were incubated in SM buffer pH 7.5 (100 mM NaCl, 8 mM MgSO4-7H2O, Tris-Cl 1 M pH7.5). All tests were performed at ruminant body temperature (38.5 °C). [file 13567_2025_1600_MOESM4_ESM.tif]

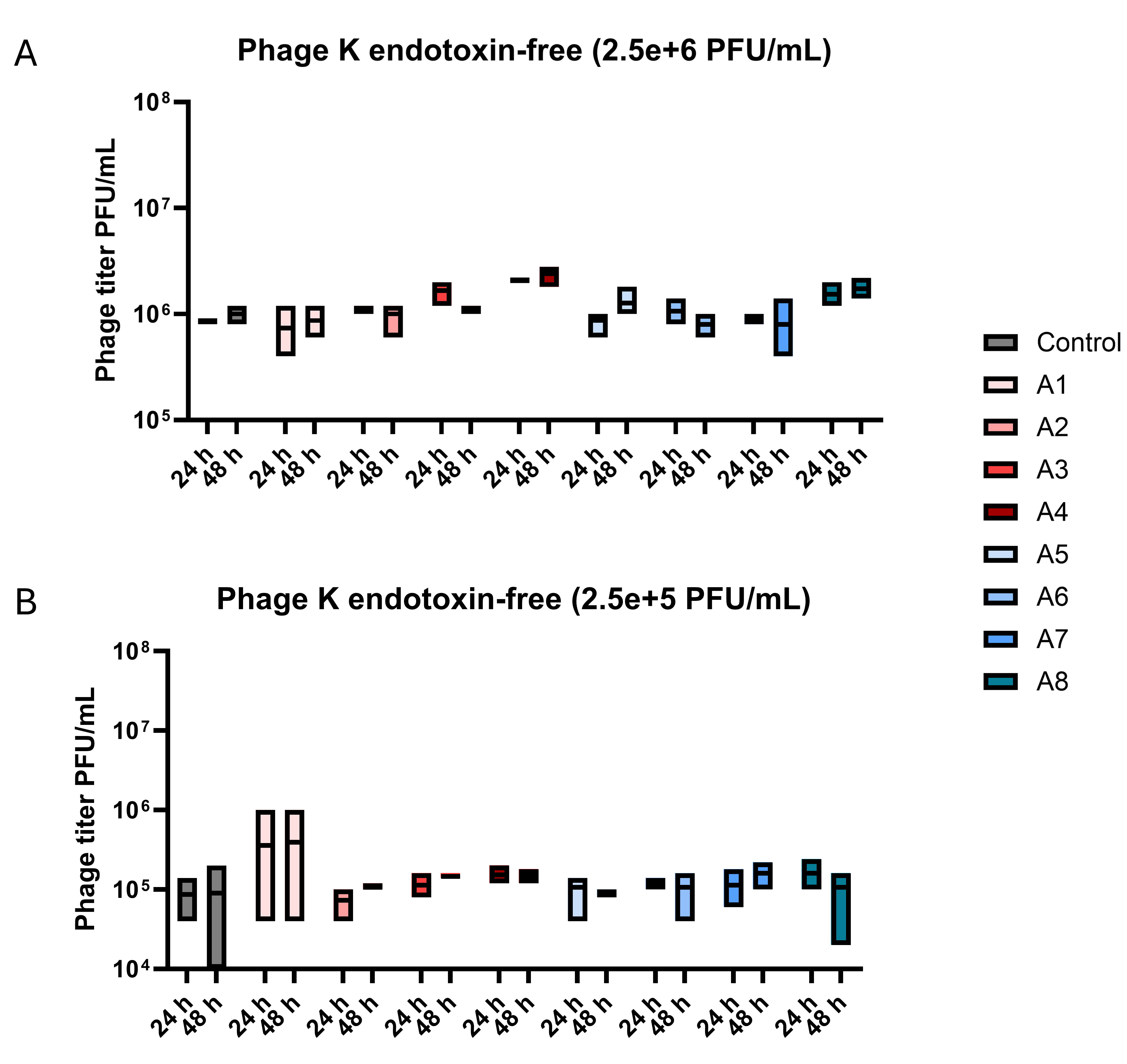

Supplement: Supplementary file 5 — Additional file 5. Bacteriophage K titers after co-incubation with PBMCs. Phage titers were determined in triplicates after 24 and 48 h of co-incubation with PBMCs. A Endotoxin-free phage K at 2.5e + 6 PFU/mL. B Endotoxin-free phage K titers after coincubation with PBMCs from each animal at 2.5e + 5 PFU/mL. Phage control stocks of K were incubated in SM buffer pH 7.5 (100 mM NaCl, 8 mM MgSO4-7H2O, Tris-Cl 1 M pH7.5). All tests were performed at ruminant body temperature (38.5 °C). [file 13567_2025_1600_MOESM5_ESM.tif]
